# Spontaneous intracranial hemorrhage as an initial manifestation of primary Sjögren’s syndrome: a case report

**DOI:** 10.1186/1471-2377-13-100

**Published:** 2013-07-29

**Authors:** Guo-Qiang Wang, Wei-Wei Zhang

**Affiliations:** 1 Department of Neurology, General Hospital of Beijing Military Region, Beijing 100700, China

**Keywords:** Sjögren’s syndrome, Vasculitis, Intracranial hemorrhage, Internal carotid artery, Moyamoya disease, Anti-Sjögren’s syndrome A antibody, Anti-Sjögren’s syndrome B antibody

## Abstract

**Background:**

Sjögren’s syndrome can involve the central nervous system; however, spontaneous intracranial hemorrhage has rarely been reported as the initial manifestation.

**Case presentation:**

We report a 39-year-old woman with primary Sjögren’s syndrome presenting with intracranial hemorrhage. The diagnosis of primary Sjögren’s syndrome was based on the presence of ocular dryness, salivary gland secretory and excretory dysfunction confirmed with dynamic tracer emission CT, and positive anti-Sjögren’s syndrome A and anti-Sjögren’s syndrome B antibodies.

**Conclusion:**

Primary Sjögren’s syndrome can present with variable central nervous system signs, which may precede the classic sicca symptoms. Therefore, Sjögren’s syndrome-associated indicators should be investigated in patients without the common risk factors for stroke who present with spontaneous intracranial hemorrhage.

## Background

Sjögren’s syndrome (SS) is a common autoimmune disease. The histopathological hallmark is periductal lymphocytic infiltration of the exocrine glands, mainly the salivary and lacrimal glands, which results in loss of secretory function. The annual incidence has been estimated at 3.9–6.0 per 100,000 population [1,2]. SS has a marked female preponderance with a female-to-male ratio of 13:1. It also features a later age at onset, with a median age of 54 years for women and 58 years for men at the first diagnosis [3]. This syndrome is classified as primary SS (pSS) in the absence of other autoimmune diseases, and as secondary SS when it is associated with other autoimmune diseases such as rheumatoid arthritis, scleroderma, systemic lupus erythematosus, AIDS, hepatitis C infection, pre-existing lymphoma sarcoidosis, graft-versus-host disease, or the use of anticholinergic drugs.

The neurological manifestations of pSS involve both the peripheral and central nervous systems. Peripheral nervous system involvement is considered the most common neurological manifestation in pSS, and is characterized by predominantly sensory or occasionally mixed neuropathies, and mononeuritis multiplex. Central nervous system involvement occurs in 5.8–68% of pSS patients [4,5] and, in most cases, neurological manifestations precede the sicca symptoms [6]. Central nervous system lesions in pSS vary from diffuse involvement, which manifests as cognitive deficits or meningoencephalitis, to focal involvement, which presents with similar symptoms to multiple sclerosis or neuromyelitis optica [7]. SS-associated infarction seldom occurs with stroke-like features such as aphasia or hemiplegia [5] and SS is even more rarely complicated with severe cerebral artery lesions, particularly hemorrhagic stroke. We report a case of a patient with pSS who presented with intracranial hemorrhage (ICH).

## Case presentation

**Figure 1:**
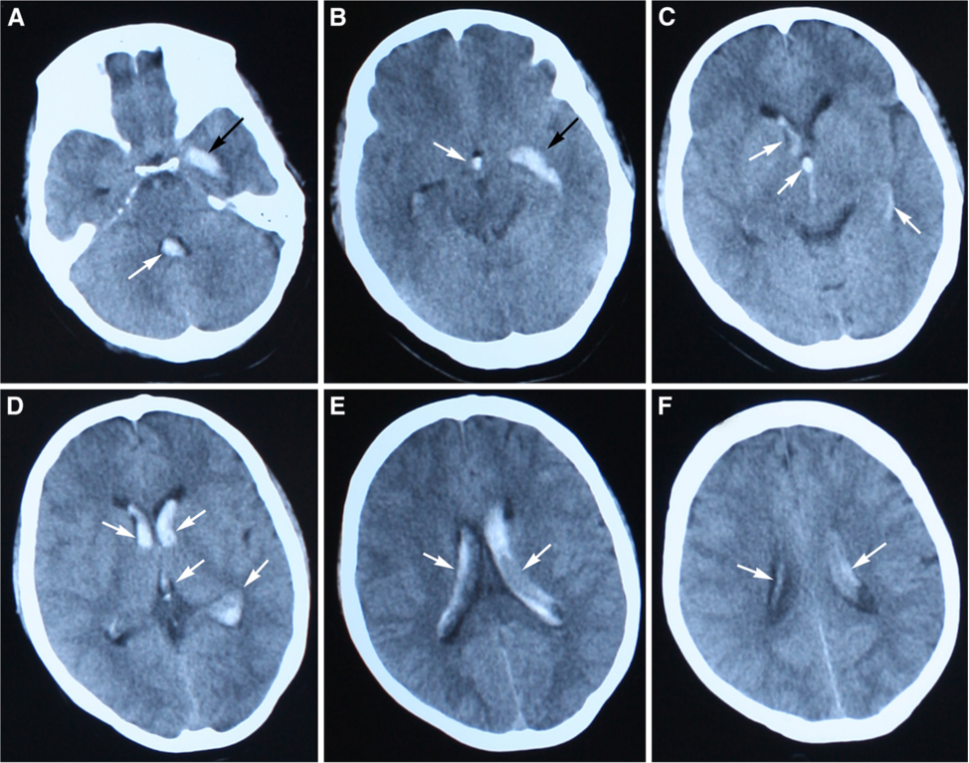
**Brain CT obtained on the day of onset.** The image shows hemorrhage in the left hippocampus **(A**, **B**, black arrow**)**, which extends into the ventricular system **(A**-**F**, white arrows**)**.

After suffering from acute headache for 2 h while washing clothes, a 39-year-old woman was admitted to the Department of Neurology, General Hospital of Beijing Military Region, in September 2012. The patient had no history of hypertension, coagulation disorders, or arthralgia, and showed no obvious xerostomia or xerophthalmia. On admission, her general examination was normal except for mild hypertension (148/90 mmHg). The neurological examination revealed positive Kernig’s sign only, without motor or sensory deficit. An urgent brain CT (Figure 1) showed hemorrhage in the left hippocampus, which extended into the ventricular system. Mannitol 250 mL was administered every 8 h for 1 week. Her headache gradually eased, the blood pressure returned to the normal range, and Kernig’s sign disappeared.

All of the additional examination results were normal, including ambulatory blood pressure and electrocardiogram; chest CT; cardiac ultrasonography; and examinations of the digestive, urinary, and uterine systems, and the breasts and appendages. Vision in the left and right eyes was 0.6 and 0.5, respectively. Schirmer’s tear test results in both eyes were <2 mm in 5 min (normal >15 mm); tear breakup time was 2 s (normal ≥10 s); and punctate fluorescein staining was >10 (normal <10).

The results of routine laboratory studies were also at normal levels, including complete blood cell count, coagulation, liver function, kidney and thyroid, lipids, glucose, glycosylated hemoglobin, C-reactive protein, and anti-O chain. Serological tests for HBsAg, hepatitis C virus, human immunodeficiency virus, syphilis, and tumor markers were negative. Erythrocyte sedimentation rate (ESR) was 62 mm/1st h; rheumatoid factor, 701 IU/mL (normal <25); antibody SS-A titer, 95 ng/mL; SS-B, 58 ng/mL; and recombinant Ro-52, 83 ng/mL (normal <10). Immunoglobulin (Ig) G was 18.7 g/L (normal 6–16); IgA, 6.86 g/L (normal, 0.4–3.3); IgM, 2.14 g/L (normal, 0.4–2.6); complement C3, 0.44 g/L (normal, 0.85–1.93); and C4, 0.13 g/L (normal, 0.12–0.36). Other auto-antibodies, including anti-β2-glycoprotein-I, anti-cardiolipin, anti-TB, and paraneoplastic antibody (anti-Hu, Yo, Ri, NMDA) were negative.

**Figure 2:**
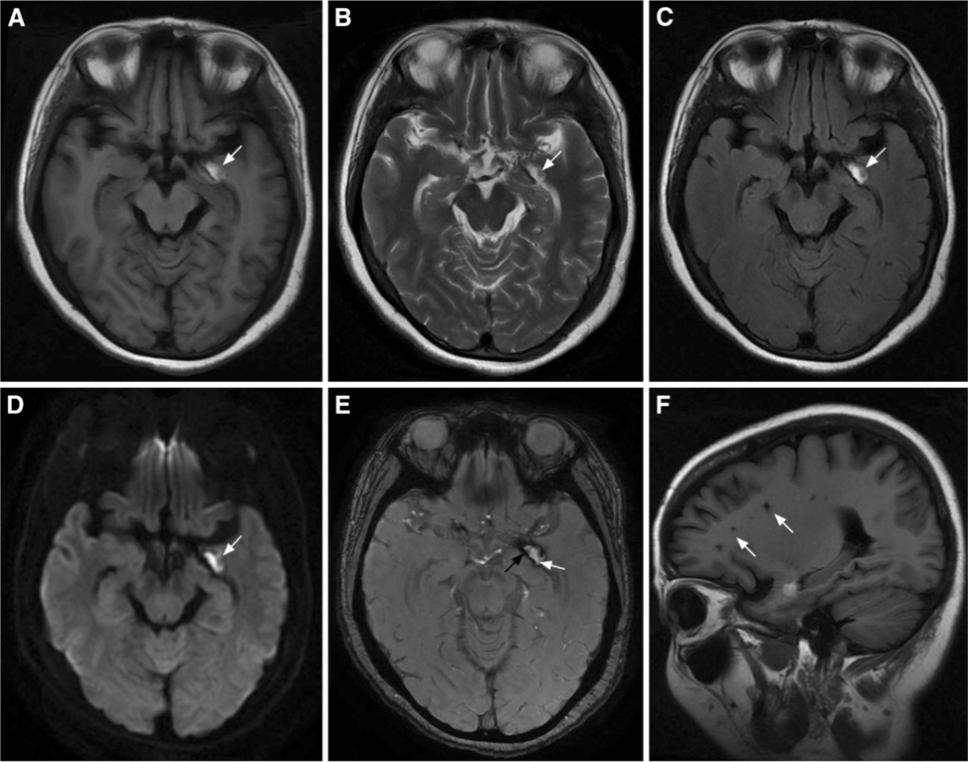
**Brain MRI obtained 5 days post-onset.** Axial T1, T2, T2-FLAIR, and DWI **(A**–**D)** show an abnormal high signal within the left lateral uncus (hippocampus) (arrows). On T2 star-weighted angiography imaging **(E)**, the lesion shows markedly low signal intensity (black arrow) with a hyperintense core (white arrow). These findings indicate that the left hippocampal hemorrhage was subacute. Sagittal T1 **(F)** shows multiple lacunar low signals in the parietal and periventricular areas (arrows), suggesting multiple lacunar infarction in the cerebral hemispheres.

Brain MRI (Figure 2) performed 5 days after admission confirmed the diagnosis of hemorrhage in the left uncus (hippocampus) with multiple lacunar infarcts in the cerebral hemispheres.

**Figure 3:**
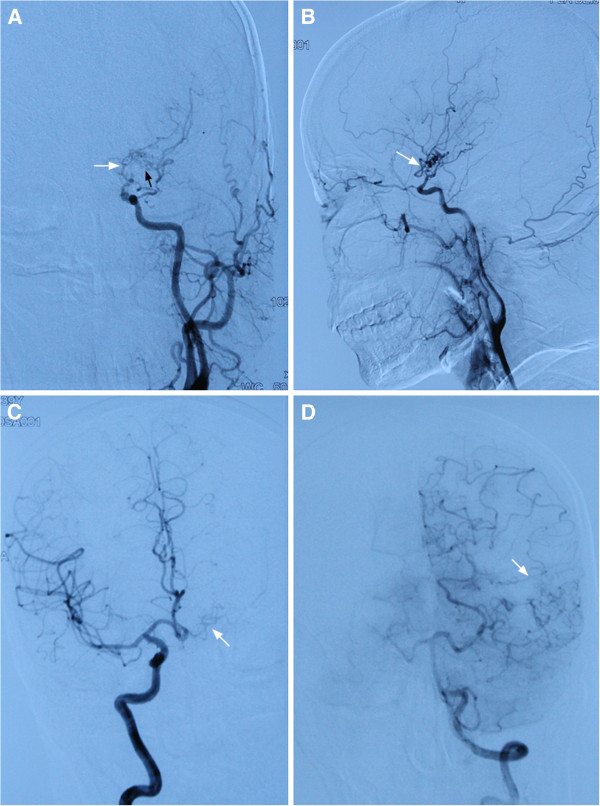
**Cerebral angiography.** The images show occlusion of the terminal portion of the left internal carotid artery **(A**, **B**, white arrow**)**. Mild collateral circulation within the left middle cerebral artery region **(C**, **D**, white arrow**)** was also seen. The collateral circulation arose from the right internal carotid artery **(C)** and the left posterior cerebral artery **(D)** to the left middle cerebral artery region. A small number of moyamoya vessels can be seen **(A**, black arrow**)**.

Cerebral angiography revealed occlusion of the terminal portion of the left internal carotid artery (Figure 3A and B). Mild collateral circulation from the right internal carotid artery (Figure 3C) and the left posterior cerebral artery (Figure 3D) to the left middle cerebral artery region were present,and moyamoya vessels were slightly seen (Figure 3A, black arrow). No aneurysm, arteriovenous malformation or obvious vascular wall irregularity was found. The cerebral venous system was not involved.

**Figure 4:**
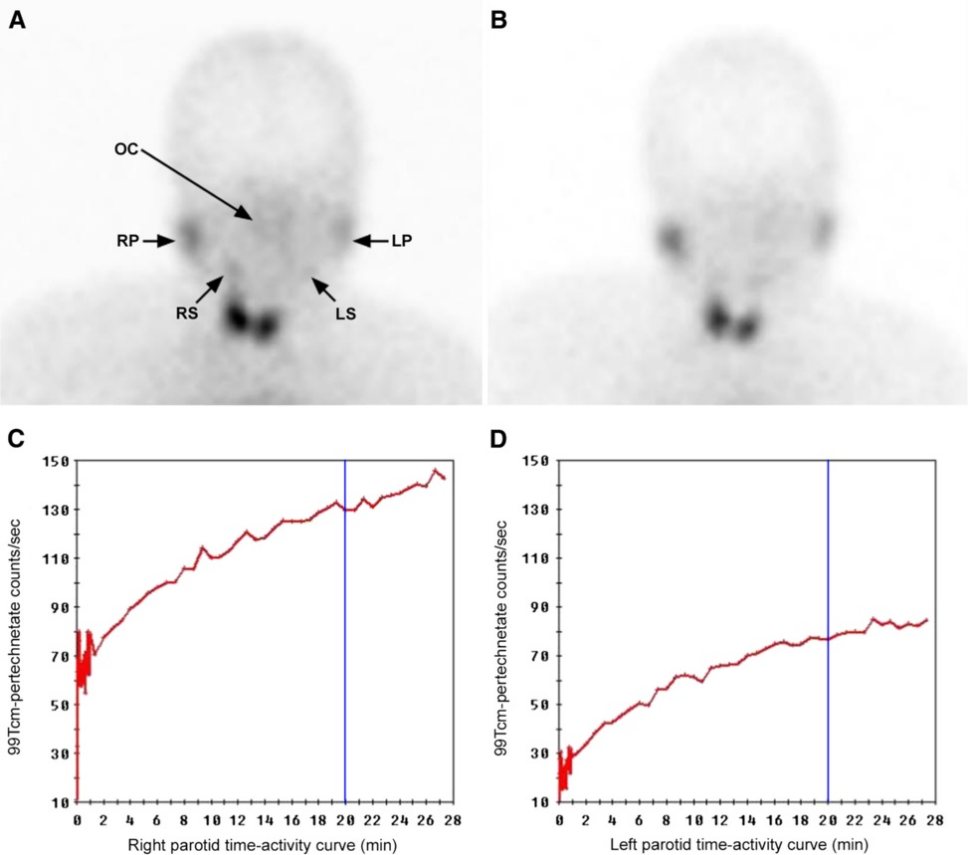
**Salivary gland dynamic tracer emission CT with 99m Tc-sodium pertechnetate.** A gustatory stimulus of vitamin C tablets begins at 20 min. The uptake capability of both submandibular and the left parotid glands is lower than that of the right parotid **(A)**. Radiotracer development after the stimulation of vitamin C tablets at 20 min showed neither obvious decline in both sets of the glands nor any visible accumulation in the oral cavity **(B)**. Parotid time-activity curve shows normal uptake on the right side **(C)**, but decreased on the left **(D)**, and no excretion on both sides after stimulation with vitamin C (blue line). OC: oral cavity; RP: right parotid gland; LP: left parotid gland; RS: right submandibular; LS: left submandibular.

Salivary gland dynamic tracer emission CT with 99mTc-sodium pertechnetate (Figure 4) showed decreased uptake in the left parotid and both submandibular glands, as well as reduced excretion in both sets of glands.

Based on the abnormal laboratory findings, the patient was diagnosed with pSS-associated cerebral vasculitis with ICH extending into the ventricular system, and with occlusion of the terminal portion of the left internal carotid artery accompanied by moyamoya vessels (quasi-moyamoya disease), as well as with multiple lacunar infarctions.

After the diagnosis, methylprednisolone 160 mg/day was administered by intravenous injection for 1 week, with oral leflunomide 10 mg/day. Follow-up CT was performed 2 weeks after admission and revealed that the hemorrhage was completely absorbed with no evidence of hydrocephalus; therefore the patient was discharged. At the 3-month follow-up, the patient was completely independent, with no discomfort, and normal physical examination findings. Laboratory tests showed positive results for SS-A/SS-B/Re-52 antibodies, and elevated rheumatoid factor, but all of the titers were lower than that of the initial results.

## Conclusion

Only five case of pSS-associated ICH have been reported [8-12]. Unlike our case, all of the five reported cases had a clear history of pSS prior to the ICH, and none had ICH as the first manifestation of pSS. Four patients developed aneurysms leading to subarachnoid hemorrhage [9-12].

Currently, vasculitis is a recognized feature of SS and is the pathological basis of the nervous system damage. Any vessels in the brain or spinal cord can be involved [5]. SS-associated systemic vasculitic manifestations occur in approximately 5–10% of SS patients [13] and two histopathological types have been suggested according to the type of the infiltrating cell: the neutrophilic type and the mononuclear cell type [14]. In SS-associated vasculitis, there is direct vessel wall invasion by neutrophilic or mononuclear cells, hyperplasia of endothelial and smooth muscle cells, and immune complex deposits. These pathological factors alter or destroy the normal structure of the vessel wall, and result in vessel lumen occlusion, or wall rupture due to changes in local pressure at a weak point in the vessel wall [14]. Abnormal collateral vessels (moyamoya vessels) at the base of the brain may develop in autoimmune diseases such as SS [8,15]. In such conditions, the abnormal vascular network is termed quasi-moyamoya disease [16]. Moyamoya vessels are prone to thrombosis or hemorrhage due to fibrin deposits in their walls, fragmented elastic laminae, attenuated media, and microaneurysm formation [17]. It is well known that hemorrhagic stroke from moyamoya vessels occurs predominantly in adult moyamoya disease patients. If hypoperfusion is confirmed, bypass surgery may be considered to prevent rebleeding [16].

The most common serologic finding in SS is hypergammaglobulinemia, which is found in 80% of pSS patients [18]. The elevated immunoglobulin levels in SS patients contain a number of autoantibodies, including rheumatoid factors, and antinuclear, anti-SS-A and anti-SS-B antibodies [3]. The presence of these autoantibodies is closely related to the occurrence of vasculitis [14].

The non-specific and diverse nature of pSS leads to an average diagnostic delay of 3–8 years [19,20], even though 100% of pSS patients have exocrine gland involvement. Patients presenting with sicca symptoms account for fewer than half of all pSS patients [20].

Our patient’s sicca symptoms were not obvious clinically either before or after hemorrhage. Currently, the diagnosis of pSS is based on the following: high titers for anti-SS-A/Ro, SS-B/La, and recombinant Ro-52 antibodies and rheumatoid factor; elevated IgG and IgA; accelerated ESR; decreased exocrine glandular function; no trace of other autoimmune or infectious disease; and the revised classification criteria for Sjögren’s syndrome proposed by the American-European Consensus Group [21]. We hypothesized that in our patient, the pSS-associated vasculitis might have contributed to the development of the asymptomatic occlusion of the left terminal portion of the internal carotid artery and the subsequent development of moyamoya vessels, ICH, and multiple lacunar infarctions.

Vasculitis-associated clues should be carefully investigated in adult female patients with stroke, particularly those without common stroke risk factors, such as hypertension or coagulopathy.

### Informed consent

Written informed consent was obtained from the patient for publication of this Case report and any accompanying images. A copy of the written consent is available for review by the Editor of this journal.

## Competing interests

We declare that we have no competing interests.

## Authors’ contributions

GQW and WWZ diagnosed and treated the patient, contributed equally to drafting and revising the manuscript. Both authors read and approved the final manuscript.

## Pre-publication history

The pre-publication history for this paper can be accessed here:

http://www.biomedcentral.com/1471-2377/13/100/prepub
